# Regulation of Hepatocyte Growth Factor in Mice with Pneumonia by Peptidases and Trans-Alveolar Flux

**DOI:** 10.1371/journal.pone.0125797

**Published:** 2015-05-04

**Authors:** Wilfred W. Raymond, Xiang Xu, Shilpa Nimishakavi, Catherine Le, Donald M. McDonald, George H. Caughey

**Affiliations:** 1 Cardiovascular Research Institute, School of Medicine, University of California San Francisco, San Francisco, California, United States of America; 2 Department of Medicine, School of Medicine, University of California San Francisco, San Francisco, California, United States of America; 3 Department of Anatomy, School of Medicine, University of California San Francisco, San Francisco, California, United States of America; 4 Northern California Institute for Research and Education, San Francisco, California, United States of America; 5 Veterans Affairs Medical Center, San Francisco, California, United States of America; Chinese Academy of Sciences, CHINA

## Abstract

Hepatocyte growth factor (HGF) promotes lung epithelial repair after injury. Because prior studies established that human neutrophil proteases inactivate HGF in vitro, we predicted that HGF levels decrease in lungs infiltrated with neutrophils and that injury is less severe in lungs lacking HGF-inactivating proteases. After establishing that mouse neutrophil elastase cleaves mouse HGF in vitro, we tested our predictions in vivo by examining lung pathology and HGF in mice infected with *Mycoplasma pulmonis*, which causes neutrophilic tracheobronchitis and pneumonia. Unexpectedly, pneumonia severity was similar in wild type and dipeptidylpeptidase I-deficient (*Dppi^-/-^*) mice lacking neutrophil serine protease activity. To assess how this finding related to our prediction that Dppi-activated proteases regulate HGF levels, we measured HGF in serum, bronchoalveolar lavage fluid, and lung tissue from *Dppi^+/+^* and *Dppi^-/-^* mice. Contrary to prediction, HGF levels were higher in lavage fluid from infected mice. However, serum and tissue concentrations were not different in infected and uninfected mice, and HGF lung transcript levels did not change. Increased HGF correlated with increased albumin in lavage fluid from infected mice, and immunostaining failed to detect increased lung tissue expression of HGF in infected mice. These findings are consistent with trans-alveolar flux rather than local production as the source of increased HGF in lavage fluid. However, levels of intact HGF from infected mice, normalized for albumin concentration, were two-fold higher in *Dppi^-/-^* versus *Dppi^+/+^* lavage fluid, suggesting regulation by Dppi-activated proteases. Consistent with the presence of active HGF, increased expression of activated receptor c-Met was observed in infected tissues. These data suggest that HGF entering alveoli from the bloodstream during pneumonia compensates for destruction by Dppi-activated inflammatory proteases to allow HGF to contribute to epithelial repair.

## Introduction

HGF is one of the most potent factors involved in lung growth and repair. Initially characterized as a mitogen for mature hepatocytes, HGF now is recognized to be pleiotropic, having motogenic, tubulogenic and anti-apoptotic effects on airway epithelial and other cells expressing HGF receptor c-Met [**1**–3]. In keeping with postulated roles of HGF in mediating interactions between epithelial and mesenchymal cells [4], mice with targeted disruption of HGF’s gene have developmental defects of lung and other organs and die before birth [5]. HGF is expressed in fetal lung mesenchyme, where it stimulates branching morphogenesis [6]. In lungs of adult mice, HGF diminishes bleomycin-induced fibroblast proliferation [7,8], suggesting a role in limiting epithelial to mesenchymal transitions leading to fibrosis. In a mouse model of asthma [9], exogenously administered HGF decreased airway remodeling associated with allergen challenge, and antagonism of endogenous HGF increased airway responsiveness to bronchoconstrictors. HGF is a mitogen and morphogen for lung epithelial and mesenchymal cells and is alternatively termed *scatter factor* because it disperses epithelial cells in culture [3]. It can be present in edema fluid in acute lung injury and is potentially produced in the lung locally by fibroblasts and inflammatory cells [10–13].

Mature HGF is a disulfide-linked, heterodimeric protein related to plasmin, with α and β chains originating from an inactive, single-chain precursor similar to plasminogen [14]. A fragment of the α chain comprised of the N-terminal hairpin and four kringle domains (NK4) antagonizes c-Met-mediated effects, such as scatter factor activity [15], and inhibits angiogenesis [16,17]. NK4 is composed of the first 447 residues of the α chain. *In vitro* studies from this laboratory showed that neutrophil and mast cell peptidases attack an inactivation segment in human HGF α chain to generate classical NK4 (1–447) and NK4-like HGF (1–449), which antagonize HGF scatter factor activity [15]. The present work identifies mouse mast cell and neutrophil proteases that generate NK4-like proteins from mouse HGF in vitro, and provides evidence that HGF levels are regulated by proteases in vivo and that influx of intact HGF from the bloodstream in mice with pneumonia potentially offsets HGF destruction by these proteases.

## Materials and Methods

### Generation of NK4-like fragments of HGF in vitro

To create a standard for comparison with mouse NK4-like fragments, human NK4 (1–449) corresponding to the protein generated from HGF by human chymase [15] was produced in High Five insect cells using a tag-free baculovirus construct (BaculoDirect, Invitrogen, Carlsbad, CA) and purified by loading onto a heparin-affinity HPLC column (Toso-Haas; Montgomeryville, PA) and eluting with a linear gradient of 0.5 to 2.0 M NaCl in 10 mM bis-Tris (pH 6.1). To determine whether mouse HGF can give rise to a similar fragment, as predicted by the high degree of sequence conservation between mouse and human HGF within the 17-residue inactivation segment [15], we incubated recombinant intact mouse HGF (R&D Systems, Minneapolis, MN) with a mouse chymase (mast cell protease 4, mMCP-4) purified from ear skin as described [18]. We also incubated mouse HGF with recombinant mouse neutrophil elastase (R&D Systems) and cathepsin G (produced as described [19]). Resulting digests were compared on Coomassie Blue-stained, non-reducing SDS-PAGE gels.

### Assay of tryptic and HGF-hydrolyzing activity of *Mycoplasma pulmonis*

To determine if the respiratory pathogen *Mycoplasma pulmonis* is a source of proteolytic and HGF-cleaving enzymes, we prepared extracts of pure washed aliquots (containing 6.2 x 10^5^ colony-forming units) of the CT7 strain of *M*. *pulmonis* that was used to infect mice in experiments described below. Bacteria were pelleted from broth at 4°C, washed twice with PBS, resuspended in 0.04 ml of 20 mM MES (pH 5.5) with 2 M NaCl, and subjected to 3 freeze-thaw cycles to release cell contents. To assess the capacity of *M*. *pulmonis* lysate to hydrolyze HGF, 2 μg of mouse HGF was incubated with the lysate for 3 hours at 37°C, after which the mixture was electrophoresed on non-reducing SDS-PAGE gels to detect products of HGF degradation. To assess general peptidase activity in the *M*. *pulmonis* extract, 1-μl aliquots of lysate were incubated separately with each of the following peptidic colorimetric substrates: tosyl-Gly-L-Pro-Lys-4-nitroanilide, benzoyl-L-Val-Gly-Arg-4-nitroanilide, succinyl-L Ala-Ala-Pro-Leu-4-nitroanilide, and succinyl-L-Val-Pro-Phe-4-nitroanilide (Sigma-Aldrich, St. Louis, MO), each at 1 mM in 0.1 ml of PBS. Hydrolysis was detected by measuring change in Absorbance at 410 nm after overnight incubation at 25°C.

### Infecting mice with mycoplasma

This study was conducted in strict accordance with the recommendations in the Guide for the Care and Use of Laboratory Animals of the National Institutes of Health. The protocol was approved by the University of California at San Francisco’s Animal Use Committee (protocol number AN104626). Specific pathogen-free mice were infected with the CT7 strain of rodent respiratory pathogen *Mycoplasma pulmonis* by methods described previously by this laboratory [20,21]. Briefly, dipeptidylpeptidase I-null (*Dppi* ^*-/-*^) and wild-type C57BL/6 (*Dppi* ^+/+^) mice were infected with *M*. *pulmonis* (0.5x10^6^ colony-forming units by intranasal instillation) to create animals with neutrophilic tracheobronchitis and pneumonia. Pilot studies showed that this dose yields moderate, non-fatal tracheobronchitis and pneumonia in both types of mice. Control mice received sterile PBS by the same route. *Dppi* ^*-/-*^ mice were obtained originally from Timothy Ley and Christine Pham of Washington University at St. Louis and were backcrossed in our laboratory at least 10 generations into the C57BL/6 background. Wild type *Dppi* ^+/+^ C57BL/6 mice were purchased from Jackson Laboratories (Bar Harbor, ME).

### Histopathological grading of tracheobronchitis and pneumonia

Mice were sacrificed up to 14 days after exposure to mycoplasma or PBS. Endpoints included body weight and pneumonia severity, which was assessed by histopathological scoring of inflammation on a 25-point scale in hematoxylin- and eosin-stained sections of lung and airway, as applied originally to hamsters [22] and adapted in this laboratory to mice infected with *M*. *pulmonis* [20].

### Bronchoalveolar lavage and lung tissue extraction

To sample the luminal contents of lungs and airways, a sterile, 22-gauge catheter was inserted into exposed tracheal lumen of anesthetized mice. Bronchoalveolar lavage fluid (BALF) was collected from three 0.8-ml aliquots of PBS per mouse and centrifuged. Supernatants were saved. Cell pellets were resuspended in equal volumes of 20-mM bis-Tris (pH 6.0) containing 2 M NaCl. In separate experimental animals, freshly excised lungs were perfused with nuclease-free PBS to remove blood. Each of the 5 lobes was bisected, with one half collected for RNA extraction and the other half collected for protein extraction. Pooled lobe halves from each mouse were snap-frozen in liquid N_2_ and stored at -80°C. For RNA extraction, frozen lung was homogenized in Trisure reagent (Bioline USA, Taunton, MA). RNA was purified from the extract via an RNeasy Plus column (Qiagen, Valencia, CA). Genomic DNA was removed by on-column digestion with DNAse I (Qiagen). For protein extraction, lobe halves were homogenized in 1 ml of 20-mM MES (pH 6). After collection by centrifugation at 20,000 x *g* for 10 min at 4°C, pellets were re-solubilized in 0.25 ml of 20-mM MES (pH 6) containing 2 M NaCl. The resulting supernatants, after centrifugation, were diluted 1:10 in 20-mM MES (pH 6) containing 2 M NaCl and 0.5% Triton X-100 for ELISA.

#### Immunoassay of HGF and albumin

HGF was quantified in BALF, lung tissue extracts and serum by ELISA (R&D Systems Mouse/Rat HGF Quantikine ELISA Kit MHG00, Minneapolis, MN) using protein standards and instructions provided by the manufacturer. Similarly, albumin was measured in BALF by mouse-specific ELISA (Genway Biotech, San Diego, CA) according to the manufacturer’s protocol.

### Measurement of HGF gene transcripts

HGF transcripts were quantified in extracts of *Dppi* ^*+/+*^ and *Dppi* ^*-/-*^ mouse lungs by reverse transcriptase-PCR using the comparative cycle threshold (C_t_) method. Briefly, mRNA extracted and purified as described above was reverse-transcribed using SensiFAST cDNA Synthesis reagent (Bioline). Quantitation of resulting cDNA was performed using PrimeTime qPCR primer sets Mm.PT.58.17970927 (mouse full–length HGF), Mm.PT.58.33540333 (mouse β-actin), and Mm.PT.a.1 (mouse glyceraldehyde phosphate dehydrogenase (GAPDH)) on the Viia7 Real-Time PCR System (Life Technologies, Grand Island, NY) using the SensiFAST SYBR No-ROX Kit (Bioline). Mice were uninfected or had been infected with *M*. *pulmonis* for one week at the time of lung harvest and extraction. Transcripts of GAPDH and β -actin genes were used to control for differences in amount of RNA extracted. Levels of HGF transcripts normalized to housekeeping gene transcripts were computed as expression ratios using comparative C_t_ (2^ΔΔCt^) values in uninfected versus infected mice of each genotype, and in *Dppi* ^*+/+*^ versus *Dppi* ^*-/-*^ mouse in the uninfected and infected groups, respectively.

### Detection of intact and fragmented HGF in vivo

Both HGF and NK4-like fragments of HGF containing the α-chain bind strongly to heparin *in vitro* [15]. To facilitate detection in dilute BALF, HGF and NK4-like fragments were captured and concentrated from BALF supernatant on heparin beads. 0.8 ml of BALF from mouse lung were rotated overnight at 4°C with 0.01 ml of heparin Affi-Gel beads (Bio-Rad, Richmond, CA), which were washed using 20 mM bis-Tris (pH 6.1) containing 0.3 M NaCl. A similar approach was used to concentrate HGF in serum. Bound proteins were released by addition of 0.56 M Tris-HCl (pH 8.5) containing 8% Li^+^ dodecyl sulfate. Unreduced samples were subjected to SDS-PAGE and electroblotted to polyvinylidene difluoride membranes (Bio-Rad).

### Immunohistochemical detection of HGF and c-Met

Formalin-fixed, paraffin-embedded tissue sections were deparaffinized in xylene, hydrated, then incubated with 3% H_2_O_2_ in H_2_O for 10 minutes to block endogenous peroxidases. Non-specific binding was blocked in 5% goat serum in Tris-buffered saline containing 0.1% Tween-20 (Sigma, St. Louis, MO)) for 1 hour at room temperature, then stained overnight at 4°C with rabbit anti-HGF (Santa Cruz Biotechnology, Santa Cruz, CA; 1:200), rabbit anti-c-Met (Santa Cruz Biotechnology; 1:50), rabbit anti-phosphorylated c-Met (Life Technologies, Grand Island, NY; 1:250), or with PBS alone as control. After 3 washes, tissue sections were incubated with goat anti-rabbit IgG-horseradish peroxidase (Santa Cruz Biotechnology; 1:3500) for 1 hour at room temperature, rewashed, then stained using a diaminobenzidine chromogen substrate detection kit (Cell Signaling Technology, Danvers, MA). Sections were counterstained with hematoxylin, dehydrated in a graded series of alcohols, cleared in xylene and mounted in Cytoseal 60 (Thermo Scientific; Pittsburgh, PA).

### Localization of c-Met by confocal microscopy

After perfusion with 1% paraformaldehyde, lungs were excised and inflated with agarose at 38°C, further fixed by immersion in 1% paraformaldehyde for 1 hour, washed for 3 hours in PBS, placed in sucrose overnight, embedded in OCT, then sliced into 50-μm sections. These sections were washed with 0.3% Triton X-100 in PBS, incubated for 1 hour in 0.3% Triton X-100/0.2% BSA/0.1% Na^+^ azide containing 10% donkey serum, then incubated overnight in 0.3% Triton X-100/0.2% BSA/0.1% Na^+^ azide containing anti-rat CD31 (Pierce; 1:500) and goat anti-c-Met (R&D Systems; 1:500). Sections then were incubated for 6 hours with donkey anti-rat IgG-Alexa Fluor-488 (Life Technologies; 1:500) and donkey anti-goat-IgG-Cy3 (Abcam; 1:500). Washed sections then were fixed in 1% paraformaldehyde in PBS, washed with PBS, and mounted in Vectashield medium with 4',6-diamidino-2-phenylindole (Vector Laboratories, Burlingame, CA).

### Measurement of protease activity in BALF

Cathepsin G-like activity in aliquots of BALF cell pellets disrupted by freeze-thawing was determined spectrophotometrically with substrate succinyl-L-Val-Pro-Phe-4-nitroanilide as described [23].

### Statistical methods

Data were compared by Student’s *t* tests or Kruskal-Wallis one-way analysis of variance, with *P* <0.05 considered to be significant.

## Results

### Generation of NK4-like protein by mMCP-4 and neutrophil elastase

**Fig 1:**
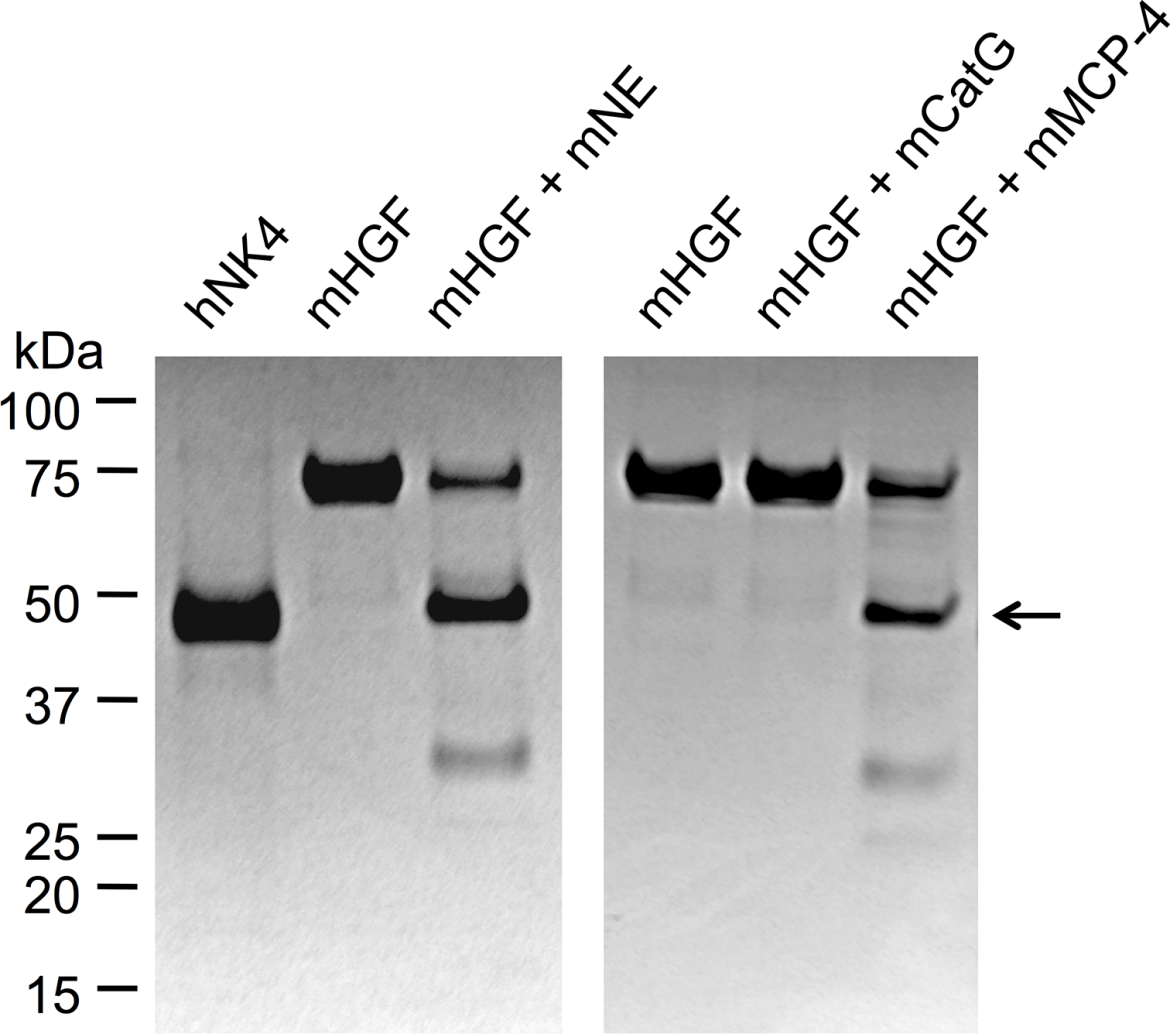
Selective proteolysis of mouse HGF generates NK4-like proteins. The images are of Coomassie-stained, non-reducing SDS-PAGE gels of mouse HGF (mHGF) incubated alone or with mouse neutrophil elastase (mNE), cathepsin G (mCatG) or mast cell chymase (mMCP-4). The leftmost lane contains human NK4 (hNK4). Migration positions of marker proteins (with molecular mass in kDa) are indicated. The arrow indicates the position of the band corresponding to NK4-like protein generated from mHGF by mNE and mMCP-4.

As shown in Fig 1, mouse chymase mMCP-4 and mouse neutrophil elastase independently hydrolyzed mouse HGF to generate a major fragment co-migrating with purified NK4-like protein generated by human chymase from human HGF. However, mouse cathepsin G lacked such activity.

### Tryptic peptidase activity but not HGF-hydrolyzing activity in *Mycoplasma pulmonis* extracts

Extracts of *M*. *pulmonis* contain low levels of tryptic peptidases cleaved the tryptic enzyme substrate tosyl-LVal-Gly-Arg-4-nitroanilide at a rate of 9 x 10^–11^ mol/min per 10^6^ colony-forming units of original *M*. *pulmonis* extract. However, *M*. *pulmonis* extracts failed to cleave tosyl-Gly-L-Pro-Lys-4-nitroanilide, succinyl-LAla-Ala-Pro-Leu-4-nitroanilide, succinyl-L-Val-Pro-Phe-4-nitroanilide, and recombinant HGF, with prolonged incubation. Therefore *M*. *pulmonis* itself is unlikely to be a source of HGF-cleaving and NK4-generating activity in the airway and alveolar lumen of *M*. *pulmonis*-infected mice.

### Inflammatory phenotype in mycoplasma-infected lungs of *Dppi* ^*-/-*^ and *Dppi* ^*+/+*^ mice

**Fig 2:**
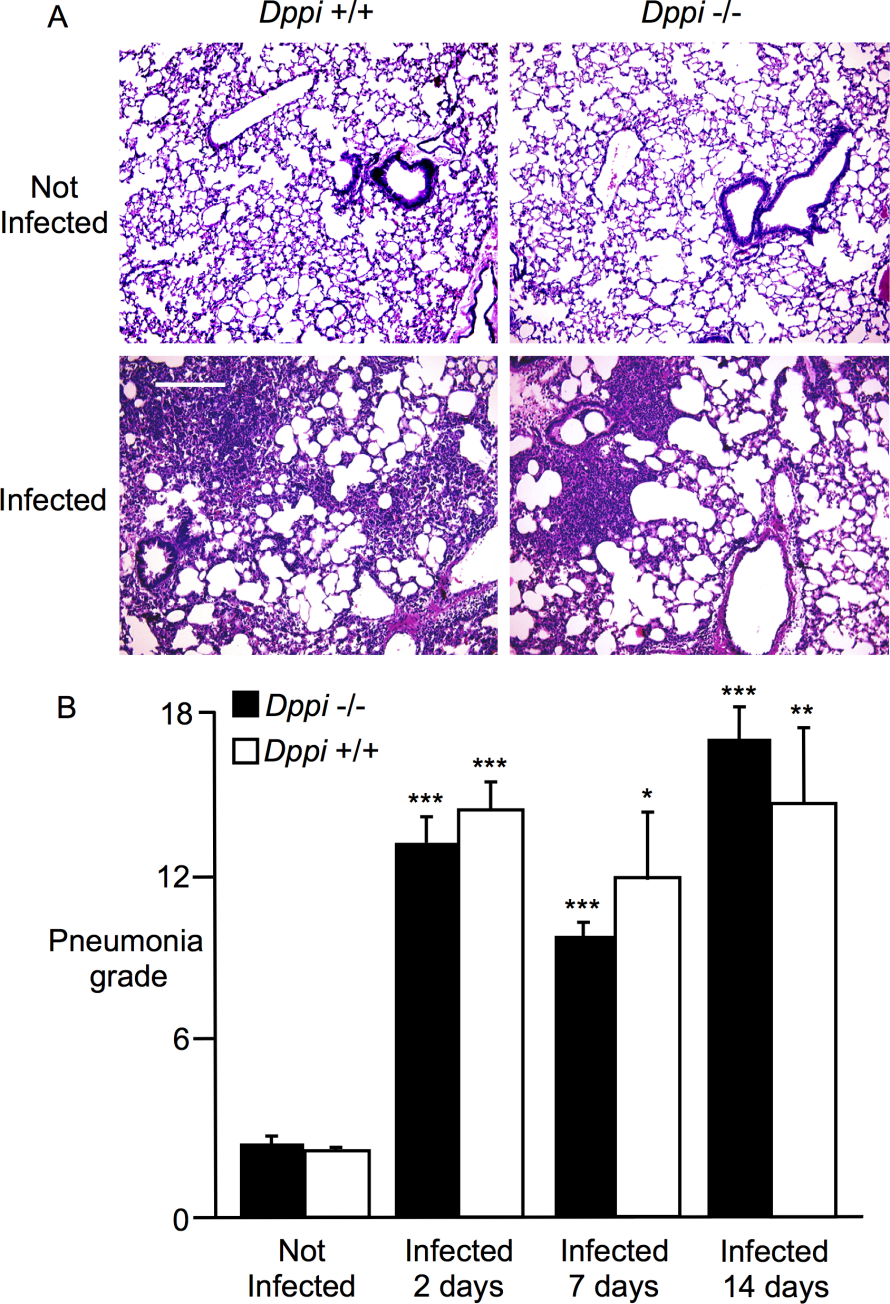
Comparison of mycoplasma tracheobronchitis and pneumonia in Dppi^-/-^ and Dppi^+/+^ mice. A: photomicrographs of hematoxylin- and eosin-stained lung sections from wild type (*Dppi* ^**+/+**^) and Dppi-deficient (*Dppi* ^**-/-**^) control mice (“Not Infected”) or mice infected 2 days previously with *Mycoplasma pulmonis*. Scale bar = 240 μm. B: comparisons of quantitative grading of airway and parenchymal inflammation (“Pneumonia grade”) in control mice (“Not Infected”) and mycoplasma-infected mice whose lungs were harvested 2, 7 and 14 days after infection; *P = 0.05, **P = 0.01, and ***P < 0.001 in comparison to “Not Infected” controls; N = 4–7 mice per group.

*Dppi* ^-/-^ and *Dppi* ^+/+^ mice developed airway and lung parenchymal inflammation after intratracheal inoculation with *M*. *pulmonis*. The inflammatory cell infiltrates were overwhelmingly neutrophilic, were present in the lumen of large and small airway, and involved airway mucosa and submucosa to a lesser degree. The lung parenchymal infiltrates were patchy in lower grades of pneumonia and more confluent in higher grades. Photomicrographs of stained lung sections from infected and uninfected *Dppi* ^-/-^ and *Dppi* ^+/+^ mice are shown in Fig 2A. Results of histopathological grading in Fig 2B reveal that tracheobronchitis and pneumonia became intense 2 days after infection in both types of mice and remained prominent, although diminished, at 7 and 14 days. At all three sampled intervals after infection, pneumonia grade was significantly higher than in uninfected mice. However, responses to infection did not differ between *Dppi* ^-/-^ and *Dppi* ^+/+^ mice.

### HGF increases in BALF but not in lung tissue or serum after infection

**Fig 3:**
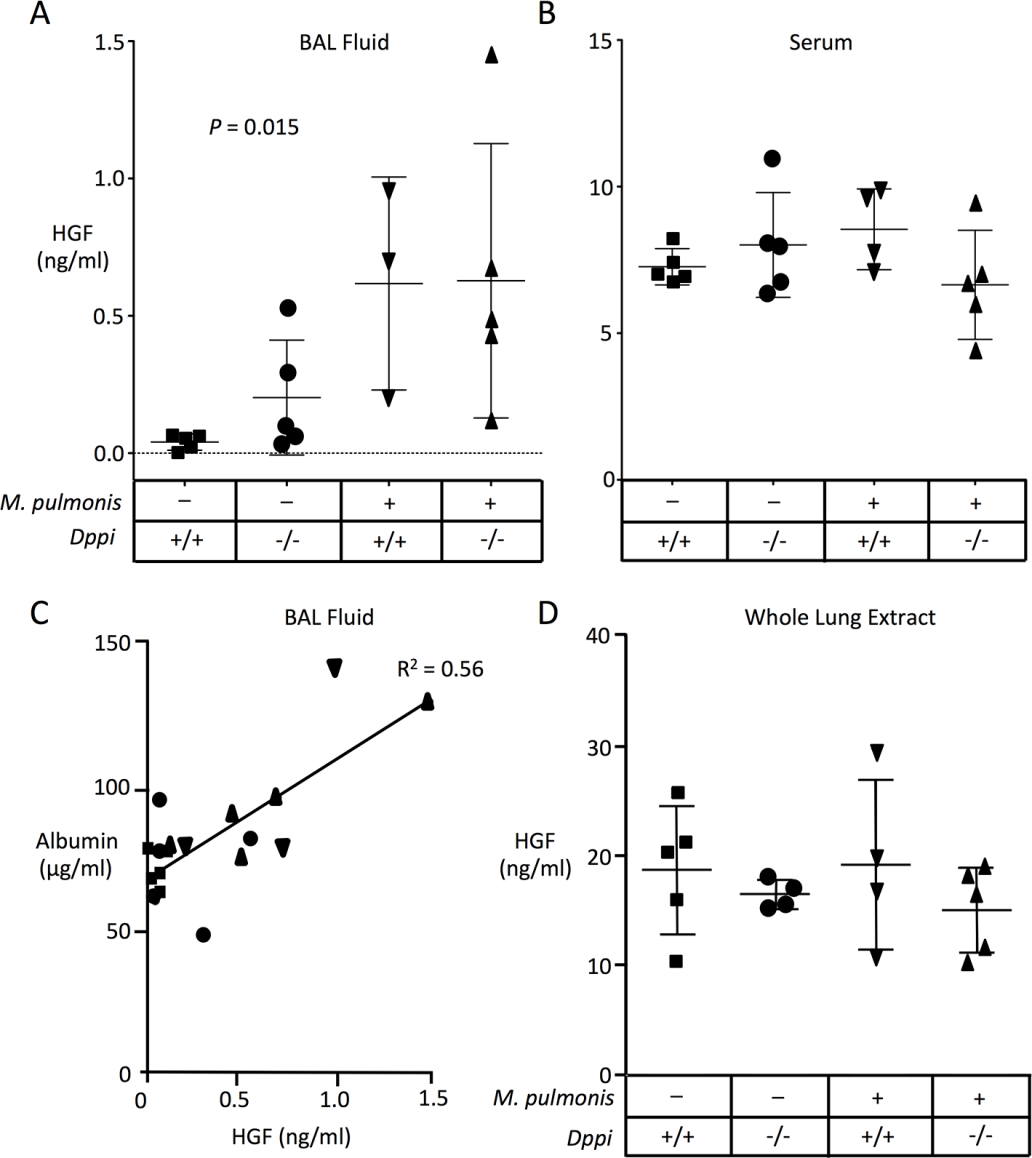
Measurement of HGF levels in BALF and serum by ELISA. Immunoreactive HGF was measured in BALF (A) and serum (B) from *Dppi* ^**-/-**^ and *Dppi* ^**+/+**^ mice that were sham-infected or *Mycoplasma pulmonis*-infected two days before acquiring samples. Bars show mean ± SD. Panel C shows relationship between HGF and albumin concentrations in paired samples of BALF. Symbols are as in panels A and B. Panel D shows results of HGF ELISA of extracts of saline-perfused whole lung. For measurements in BALF, P = 0.015 as assessed by Kruskal Wallis one-way ANOVA. Differences in levels of HGF in serum and whole lung extracts were not significant.

As shown in Fig 3A, levels of HGF assessed by ELISA increased approximately 15-fold in *Dppi* ^-/-^ and *Dppi* ^+/+^ mice after infection compared to baseline in uninfected *Dppi* ^+/+^ mice. HGF levels also were somewhat higher in uninfected *Dppi* ^-/-^ mice than in *Dppi* ^+/+^ mice, consistent with a role for Dppi-activated proteases in regulating baseline levels of immunoreactive HGF in BALF. However, as shown in Fig 3B and 3D, no significant differences in levels of immunoreactive HGF were seen in serum or lung tissues between uninfected and infected groups of mice. In uninfected *Dppi* ^+/+^ mice, the mean concentration of HGF was 195-fold higher in serum than in BALF. However, in infected *Dppi* ^+/+^ mice, the difference was only 14-fold.

### HGF and albumin increase in parallel in BALF after infection

As shown in Fig 3C, rises in HGF in BALF, as detected by ELISA, correlated with increases in albumin in BALF. The increases in albumin were most striking in infected mice, consistent with mycoplasma-associated inflammation causing leakage of HGF and albumin from the bloodstream.

### Comparative expression of HGF mRNA in lung decreases after infection

No substantial differences in HGF transcripts in lung extracts were detected between uninfected and infected mice, or between *Dppi* ^*+/+*^ and *Dppi* ^*-/-*^ mice, after normalization for housekeeping gene expression (4–5 mice per group). For example, using β-actin transcripts to control for differences in mRNA extraction, the expression ratio (2^ΔΔCt^) of HGF transcripts was 0.90 ± 0.19 (mean ± SD) in infected *Dppi* ^*+/+*^ lungs relative to uninfected *Dppi* ^*+/+*^ lungs, 0.73 ± 0.39 in uninfected *Dppi* ^*-/-*^ lungs relative to uninfected *Dppi* ^*+/+*^ lungs, 1.76 ± 1.01 in infected *Dppi* ^*-/-*^ lungs relative to infected *Dppi* ^*+/+*^ lungs, and 1.20 ± 0.70 in infected *Dppi* ^*-/-*^ lungs relative to infected *Dppi* ^*+/+*^ lungs.

### Increased levels of intact HGF in BALF after infection

**Fig 4:**
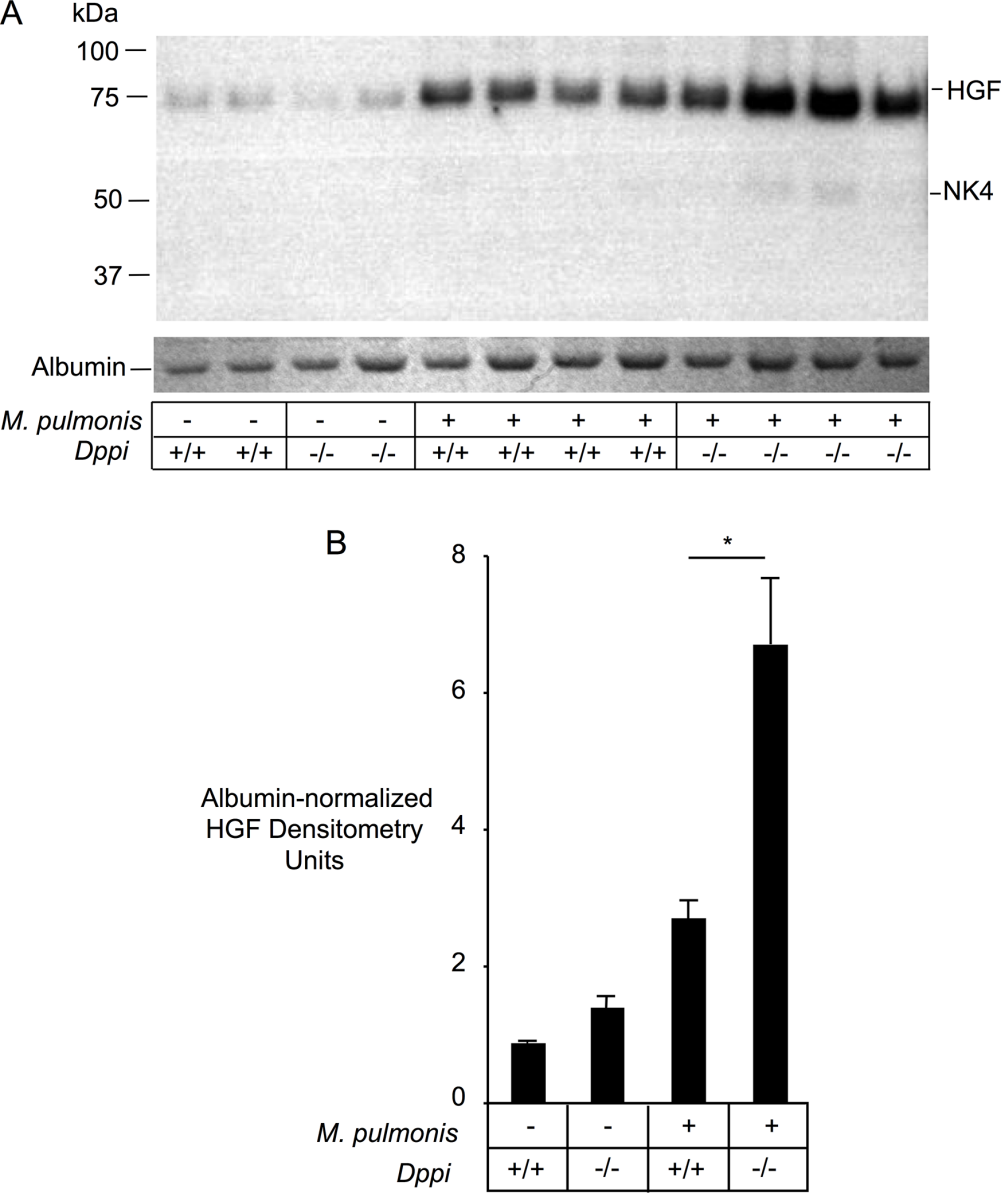
HGF and NK4-like fragments in *Mycoplasma pulmonis*-infected Dppi^-/-^ and Dppi^+/+^ mice. Panel A shows anti-HGF immunoblots of BALF proteins in parallel with albumin bands from a Coomassie blue-stained SDS-PAGE gel of the same samples. The panel also shows immunoblots of HGF in serum collected 2 and 7 days after mycoplasma infection. Panel B shows results of densitometry of HGF bands normalized to albumin band intensity. Infected mice were lavaged 24 hours after receiving a nasal inoculum of 0.5x10^**6**^ *M*. *pulmonis*.

To determine whether the increase in levels of BALF HGF detected by ELISA in infected mice was due to influx of intact HGF or due to enhanced generation of proteolytic fragments with preserved epitopes, we compared HGF’s electrophoretic behavior in BALF supernatants. Immunoblotting of proteins from non-reducing SDS-PAGE gels distinguished between intact HGF and partially degraded fragments, including NK4-like protein. Levels of intact HGF were analyzed by densitometry after normalization to the density of albumin bands in the same samples to control for differences in BALF dilution and differences in influx of serum proteins similar to HGF in size (albumin ~66 kDa versus intact HGF ~72 kDa). As shown in Fig 4, levels of intact HGF were 3.1-fold higher in BALF from infected *Dppi* ^+/+^ mice than from uninfected *Dppi* ^+/+^ mice, and 4.8-fold higher in *Dppi* ^-/-^ mice. These findings suggest that mycoplasma infection raised levels of intact HGF in lungs of infected mice. Furthermore, levels of albumin-normalized, intact HGF were 1.6-fold and 2.5-fold higher, respectively, in uninfected and infected *Dppi* ^-/-^ mice than in corresponding uninfected and infected *Dppi* ^+/+^ mice, consistent with a role for Dppi-activated peptidases in reducing levels of intact HGF, with or without infection. By far the majority of HGF was uncleaved, suggesting that most HGF detected in BALF by ELISA is intact HGF, rather than protease-generated inactive fragments. However, as shown in Fig 4, light bands corresponding to NK4-like fragments were detected in BALF from mycoplasma-infected mice, suggesting that processing of HGF into NK4-like fragments occurs in vivo.

### Tissue-selective expression of HGF and c-Met

**Fig 5:**
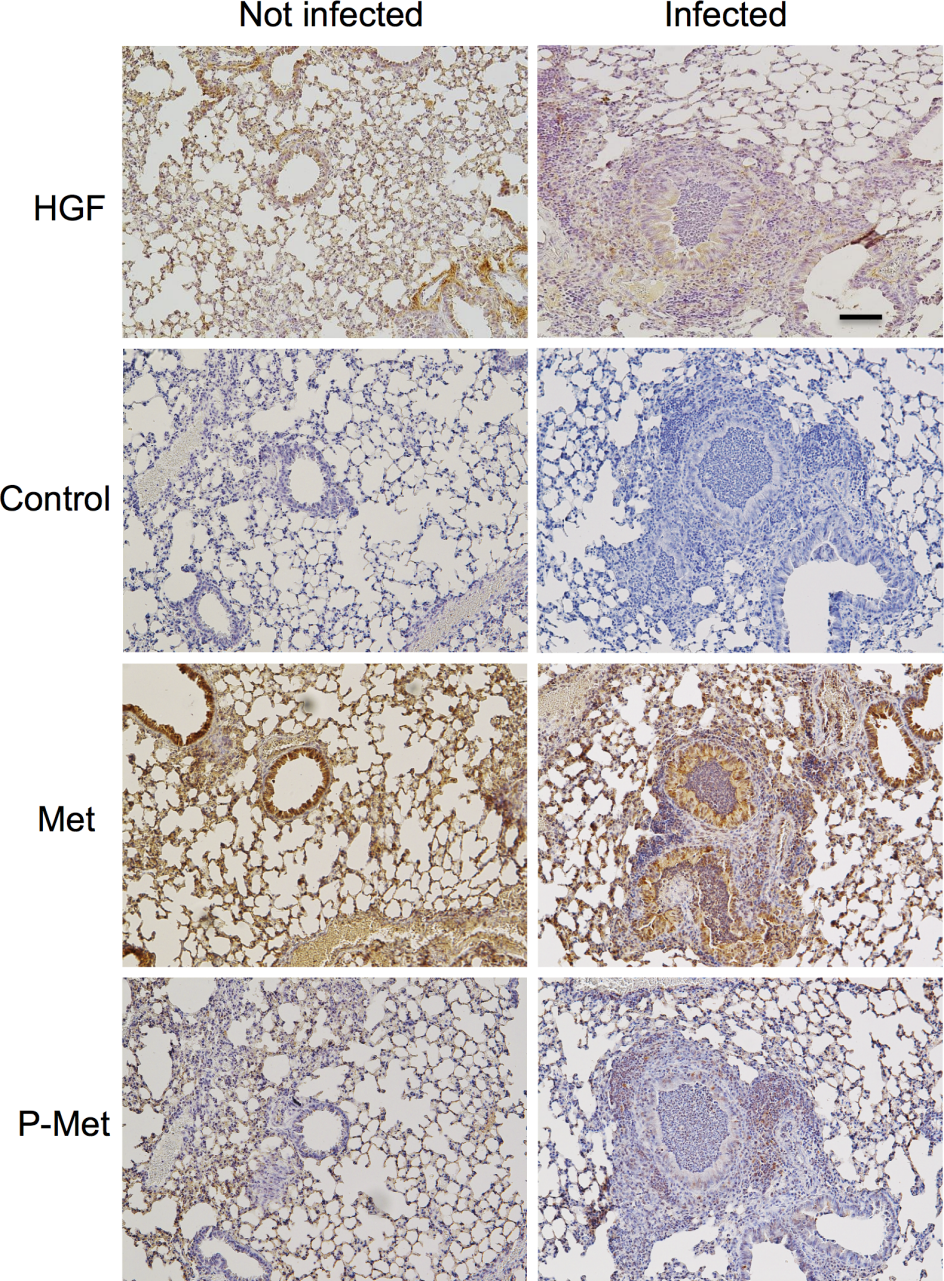
Lung and airway expression of HGF and HGF receptor c-Met. Representative low-power images of lung sections were obtained from sham-infected mice (left panels) or from mice 7 days after initial infection with *M*. *pulmonis* (right panels) after immunostaining for HGF, c-Met or activated phospho-c-Met. Control sections were stained like the other sections but with omission of primary antibody. Scale bar = 100 μm.

**Fig 6:**
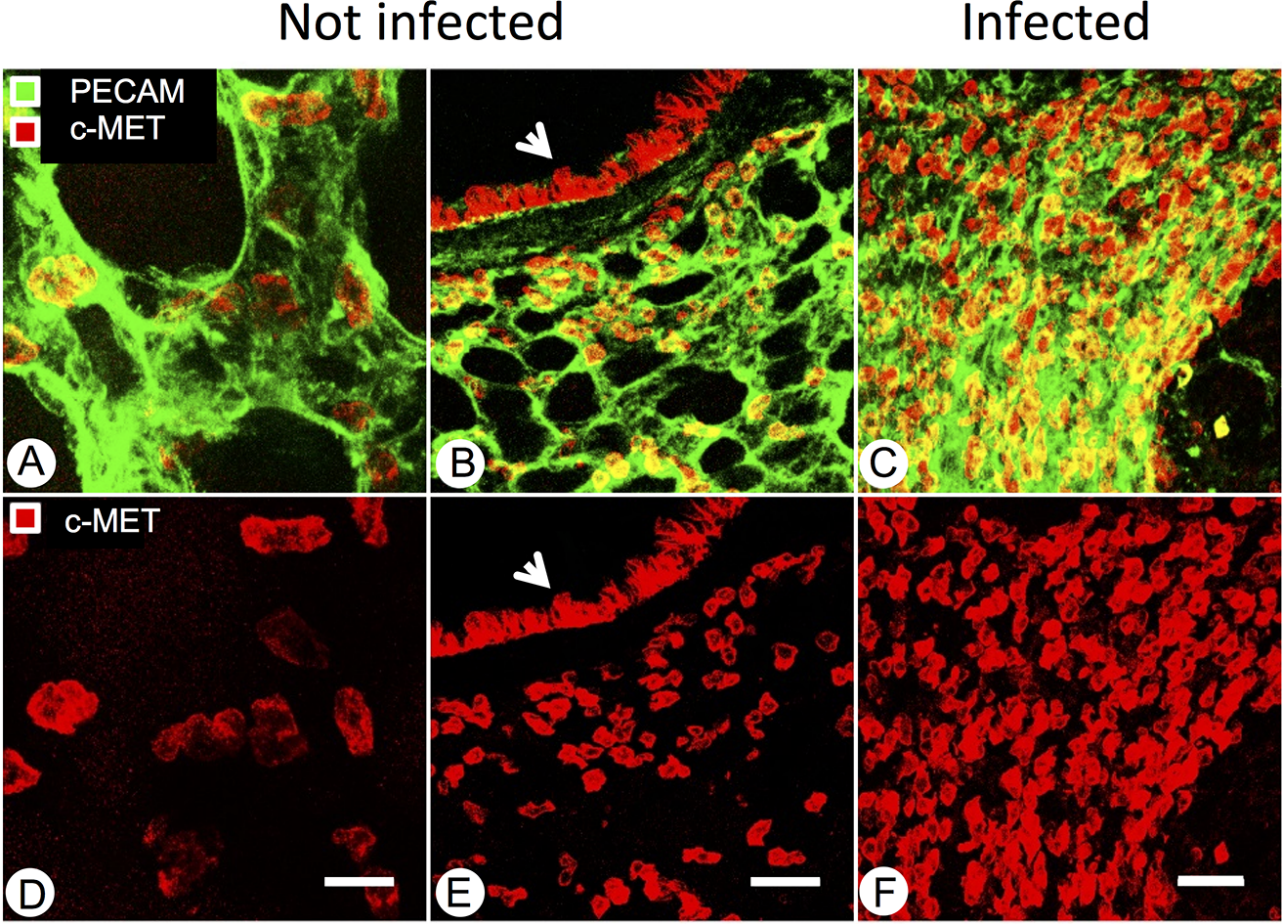
Localization of c-Met expression by confocal immunofluorescence microscopy in pathogen-free and mycoplasma-infected lungs. A: representative image of endothelial cell (green, PECAM-1) and c-MET (red) staining of alveoli in a pathogen-free mouse. Scale bar = 20 μm. B: Representative image of PECAM-1 (green) and c-Met (red) staining in lung section of a pathogen-free mouse. Airway epithelium is marked by an arrowhead. Scale bar = 50 μm. C: representative image of PECAM-1 (green) and c-Met (red) staining in lung section of a 7-day *M*. *pulmonis*-infected mouse. Scale bar = 50 μm. D, E, and F: c-Met staining only, corresponding to A, B, and C, respectively.

To identify and compare local sources of HGF and of its receptor c-Met in pathogen-free and mycoplasma-infected mice, lung tissue sections were immunostained with antibodies detecting HGF, c-Met and activated phospho-c-Met (Fig 5). HGF expression was diffuse but similar in airway and alveolar epithelial structures in pathogen-free and infected mice. No non-specific staining was seen in sections incubated with secondary antibody alone, omitting primary antibody. c-Met immunoreactivity was strong in airway epithelium but weak to absent in stromal tissues. Phospho-c-Met staining was lighter but with similar overall distribution to that of c-Met in epithelial structures from pathogen-free and infected mice. However, compared to tissues from pathogen-free mice, lungs from infected mice were infiltrated by inflammatory cells, some of which were recognized by antibodies to c-Met and phospho-c-Met. Therefore, overall expression of activated c-Met increased in infected lungs. To localize expression of c-Met in airway and alveolar structures in relation to vascular structures, lung tissues were subjected to confocal immunofluorescence microscopy using antibodies to c-Met and to endothelium-selective CD31. These results, shown in Fig 6, confirm strong expression of c-Met in airway epithelial cells and localize alveolar expression to a subset of epithelial cells and intra-alveolar mononuclear cells, in which expression increased in lungs from infected mice. c-Met was not detected in alveolar endothelium, as indicated by the lack of co-localization of the c-Met signal with CD31.

## Discussion

**Fig 7:**
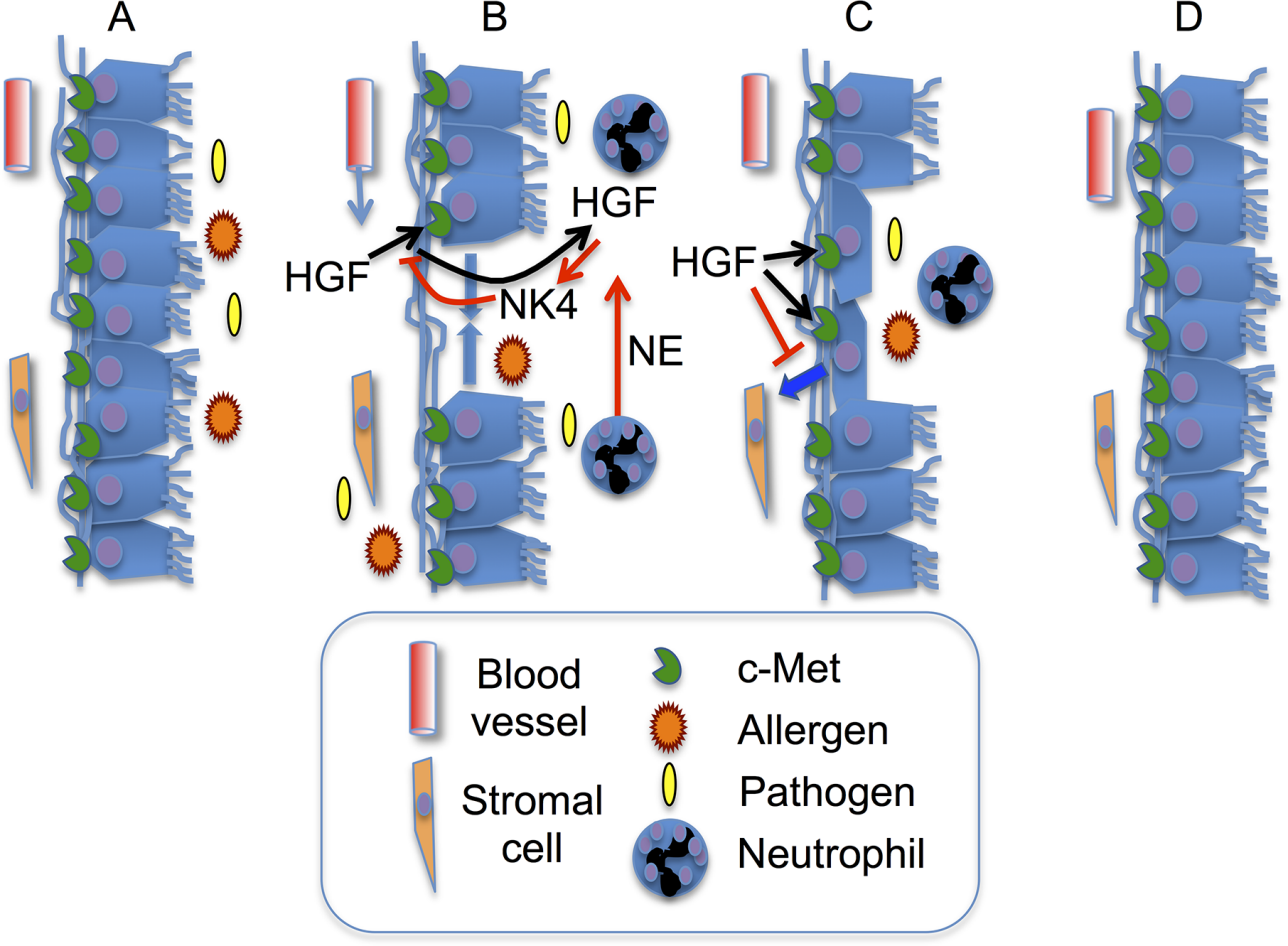
Hypothesized roles of HGF and neutrophil peptidases in epithelial repair. A: Airway epithelium is exposed to allergens, bacteria or other pathogens. B: HGF moves from bloodstream into airway via leaky and damaged endothelial and epithelial barriers. Some HGF is inactivated by neutrophil proteases, such as elastase (NE, red arrow), thereby generating HGF fragment NK4, which interferes with HGF activation of receptor c-Met. C: HGF blocks epithelial to mesenchymal transformation (blue arrow) and stimulates epithelial cell migration to repair damaged epithelium. D: Inflammation resolves and epithelial barrier is restored.

The present results, combined with prior data published by our laboratory and others, allow us to envision several modes of regulation to influence HGF’s contributions to airway epithelial repair in response to inflammatory injury. These pathways are schematized in Fig 7. We propose that proteolytic inactivation of HGF and generation of NK4-like antagonists is productive or counter-productive, depending on context. Extensive destruction of HGF and NK4 generation could prevent or delay epithelial repair and promote epithelial to mesenchymal transformation (which HGF opposes), leading to scarring and enduring epithelial dysfunction. Thus, the overall increase in intact HGF in BALF from mycoplasma-infected may assist in recovery from pathogen-associated injury. On the other hand, modulation of HGF activity by proteolytic inactivation and NK4 generation could be beneficial during the early phases of active infections by preventing premature repair while the immune system is engaged in processing and presenting antigen, raising antibodies, and recruiting immune effector proteins and cells from the bloodstream to defend against invading organisms.

The tissue origins of the observed increase in HGF levels in BALF from infected lungs are probably multiple. Some of the production is undoubtedly local, given the immunohistochemical evidence of expression in alveolar and airway structures. However, because overt differences in lung tissue expression were not observed between infected and specific pathogen-free mice, the increase above baseline of HGF levels in BALF retrieved from infected lungs may be extrapulmonary in origin. The latter possibility is supported by the observed parallel increase in BALF albumin, which likely enters airspaces from capillaries and interstitial fluid via alveolar walls damaged by infectious inflammation. An extrapulmonary, intravascular origin of the HGF in BALF is also supported by the lack of a difference between uninfected and infected mice in HGF levels in extracts of lungs after perfusion to remove the majority of intravascular contents. Although peripheral blood neutrophils are potential sources of secretable HGF [10], there was little immunostaining of neutrophils in sections of infected mouse lung. This may be because neutrophil expression is scant relative to sources like epithelium. Indeed, little HGF mRNA has been detected in human neutrophils [10].

This study reveals fragmentation of HGF into NK4-like proteins in mice with mycoplasma-induced neutrophilic tracheobronchitis and pneumonia, thereby providing *in vivo* evidence of HGF antagonist generation in lung tissues. The possibility of generating an HGF antagonist from HGF itself was explored initially using pancreatic elastase to produce a fragment with which to help establish structure-activity relationships between domains [16]. The possible contribution of splice variants of the HGF gene in generating antagonistic fragments also has been explored [24,25]. The major pancreatic elastase-generated fragment (termed NK4 because it contains HGF’s N-terminal domain and all 4 kringle domains), has anti-tumor and anti-angiogenic activity in mice when directly injected or when generated by infection with an NK4-expressing adenoviral vector [26–28]. Subsequent studies by our laboratory showed that purified human mast cell and neutrophil peptidases (which are more likely than pancreatic elastase to encounter HGF in inflamed lung tissues) have similar capability *in vitro* [15], giving rise to the hypothesis that generation of NK4-like antagonists occurs *in vivo*. Support for this hypothesis was offered by a recent study identifying NK4-generating activity in neutrophil-rich exudates from non-healing wounds in human skin [29]. The present study tested this hypothesis in mice by exploring selective hydrolysis of mouse HGF by mouse mast cell- and neutrophil-derived proteases *in vitro* and by examining HGF and NK4-like fragments in protease-deficient mice with and without pneumonia. Our finding that *Dppi* ^-/-^ mice deficient in neutrophil elastase [30] have higher lung levels of intact HGF than wild-type mice (when both kinds of mice have pneumonia) supports the hypothesis that neutrophil elastase or other DPPI-activated proteases regulate levels of HGF in neutrophilic lung and airway inflammation. The findings also suggest that NK4-like antagonist generation is at least partly offset by increased production of intact HGF, as is consistent with prior studies suggesting that local levels of HGF in mouse lung can rise in response to an inflammatory stimulus such as allergen challenge [31,32]. Thus, our study also reveals for the first time that local levels of HGF in lung and airway rise in response to bacterial infection. The observed strong increases in lung levels of HGF may compensate for enhanced peptidolytic inactivation and endogenous generation of NK4-like antagonists to allow HGF to moderate inflammation and remodeling and contribute to epithelial repair [9,33,34].

Our results establish that the early response to intratracheal inoculation with *M*. *pulmonis*, as reflected by histopathological grade of tracheobronchitis and pneumonia, does not differ in *Dppi* ^+/+^ versus *Dppi* ^-/-^ mice. This suggests that Dppi, despite its importance in activating neutrophil serine peptidases like cathepsin G and elastase [30] and the demonstration of defective neutrophil responses and killing of some bacteria in cathepsin G and neutrophil elastase-deficient mice [2,35–37], does not overtly influence the severity or evolution of neutrophilic inflammation during the first 2 weeks of infection. This argues against a major, non-redundant role for Dppi (or for neutrophil serine proteases like cathepsin G and elastase that depend on Dppi for activation) in recruitment or migration of neutrophils in response to mycoplasma infection. It should be noted that except in mice with severe immune deficits [38], *M*. *pulmonis* organisms—although they can cause fatal tracheobronchitis and pneumonia [20,21]—are thought to remain extracellular and to be contained within the lumen of the respiratory tract. Recruitment of neutrophils into the airway, and the associated angiogenesis, lymphangiogenesis and epithelial remodeling, results from formation in the airway lumen of complexes between mycoplasma antigens and antibodies raised during the adaptive immune response to mycoplasma infection [38]. Therefore, destruction of mycoplasma antigens and antigen-antibody complexes by proteases may accelerate resolution of inflammation and reduce recruitment of neutrophils in the airway. It also is possible that neutrophil proteases activate, destroy or stimulate production of bacterial proteins that modulate the inflammatory response [39]. However, our finding that inflammation is similar in *Dppi* ^+/+^ and *Dppi* ^-/-^ mice 2, 7 and 14 days after infection suggests that Dppi-activated proteases, including neutrophil cathepsin G, do not destroy mycoplasma antigens or immunomodulatory factors to an extent that lessens pneumonia severity during early responses to infection.

Past investigations from this laboratory showed that mast cells modulate host responses to airway mycoplasma infection [20]. Mice lacking mast cells develop higher burdens of lung and airway bacteria and more severe pneumonia than wild type mice after *M*. *pulmonis* infection, and are more likely to die [20]. Like neutrophils, mast cells contain Dppi-activated serine proteases, notably chymases, with potential roles in regulating inflammatory responses to bacteria. In *Dppi* ^-/-^ mice, mast cell chymase activity is low or undetectable [40]. The present finding that pneumonia grade is similar in wild type and Dppi-deficient mice argues against a major role for chymases in mediating mast cell modulation of airway inflammatory responses to mycoplasma infection. Nonetheless, as shown in Fig 1, the mouse chymase mMCP-4, like its functional homologue in humans [15], generates an NK4-like antagonist of HGF scatter factor activity *in vitro*. Although the present studies suggest that mMCP-4 and related chymases potentially contribute to the observed decrement in intact HGF in *Dppi* ^-/-^ mice relative to *Dppi* ^+/+^ mice, Dppi-activated neutrophil proteases likely are more important, given the overwhelming numerical superiority of neutrophils in mycoplasma-infected lung [20,21]. Intriguingly, mouse cathepsin G, unlike its human counterpart, does not readily cleave HGF in vitro. This may be because mouse cathepsin G is much more restricted in primary specificity than is human cathepsin G [19]. This result leads us to predict that elastase and related elastolytic proteases are more important than cathepsin G in degrading HGF in mice.

In summary, the present work provides evidence that Dppi-activated leukocyte proteases regulate levels of HGF, while suggesting that this effect may be offset in the context of bacterial pneumonia by influx of HGF from the blood.
